# Matrix stiffness induces Drp1-mediated mitochondrial fission through Piezo1 mechanotransduction in human intervertebral disc degeneration

**DOI:** 10.1186/s12967-023-04590-w

**Published:** 2023-10-10

**Authors:** Wencan Ke, Bingjin Wang, Zhiwei Liao, Yu Song, Gaocai Li, Liang Ma, Kun Wang, Shuai Li, Wenbin Hua, Cao Yang

**Affiliations:** Department of Orthopaedics, Union Hospital, Tongji Medical College, Huazhong University of Science and Technology, Wuhan, 430022 China

**Keywords:** Matrix stiffness, Mechanotransduction, Mitochondrial fission, Piezo1, Nucleus pulposus, Intervertebral disc degeneration

## Abstract

**Background:**

Extracellular matrix stiffness is emerging as a crucial mechanical cue that drives the progression of various diseases, such as cancer, fibrosis, and inflammation. The matrix stiffness of the nucleus pulposus (NP) tissues increase gradually during intervertebral disc degeneration (IDD), while the mechanism through which NP cells sense and react to matrix stiffness remains unclear. In addition, mitochondrial dynamics play a key role in various cellular functions. An in-depth investigation of the pathogenesis of IDD can provide new insights for the development of effective therapies. In this study, we aim to investigate the effects of matrix stiffness on mitochondrial dynamics in IDD.

**Methods:**

To build the gradient stiffness model, NP cells were cultured on polystyrene plates with different stiffness. Western blot analysis, and immunofluorescence staining were used to detect the expression of mitochondrial dynamics-related proteins. Flow cytometry was used to detect the mitochondrial membrane potential and intracellular Ca^2+^ levels. Apoptosis related proteins, ROS level, and TUNEL staining were performed to assess the effect of substrate stiffness on NP cells.

**Results:**

Stiff substrate increased phosphorylation of dynamin-related protein 1 (Drp1) at Ser616 by activating extracellular signal-regulated kinase 1/2 (ERK1/2) pathway, which promoted mitochondrial fission and apoptosis in NP cells. Furthermore, Piezo1 activation was involved in the regulation of the post-translational modifications of Drp1 and mitochondrial fission caused by matrix stiffness. Inhibition of Piezo1 and ERK1/2 can effectively reduce stiffness-induced ROS elevation and apoptosis in NP cells.

**Conclusions:**

Our results revealed that stiff substrate causes Piezo1 activation and Ca^2+^ influx, results in ERK1/2 activation and phosphorylation of Drp1 at S616, and finally leads to mitochondrial fission and apoptosis in NP cells. These findings reveal a new mechanism of mechanotransduction in NP cells, providing novel insights into the development of therapies for treating IDD.

**Supplementary Information:**

The online version contains supplementary material available at 10.1186/s12967-023-04590-w.

## Introduction

Low back pain (LBP) is a major cause of chronic disability and imposes a significant economic burden worldwide [1]. Intervertebral disc degeneration (IDD), leads to structural disorders and inflammatory responses of spine, and has been identified as the leading contributor of LBP [2, 3]. IDD is a multifactorial pathological process, resulting from such causes such as biological alterations, mechanical wear, and loss of nutrition [4, 5]. Various stimuli induce nucleus pulposus (NP) cells damage and senescence, leading to the development of IDD. Currently, there is no acceptable model to explain the pathogenesis of this disabling disease. Therefore, an in-depth investigation of the pathogenesis of IDD can increase our understanding of this disease and provide new insights for the development of effective therapies.

There is increasing evidences that suggest the pathological changes, caused by mechanical stimulation of spinal movement, are the primary factor of IDD. The mechanical stimuli of the intervertebral disc including compressive, tensile, and shear stresses [6–8]. The effects of compression stress on NP cells have been widely investigated among these factors. For example, several studies have reported that compressive loads increased the expression of catabolic genes (MMP-3, -13, ADAMTS-4) and extracellular matrix (ECM) degradation [9, 10]. We previously demonstrated that compression stress induced senescence and apoptosis of NP cells during IDD by regulating cytoskeleton remodeling and autophagy [11, 12]. Another important mechanical cue involved in the IDD process is the increase in NP tissues stiffness. Matrix stiffness significantly regulates cell behavior, including proliferation, migration, and differentiation [13, 14]. Over the past few decades, several strategies were developed to modulate substrate stiffness by adjusting the composition or the molecular structure [15]. Among them, alginate, hyaluronic acid, silk fibroin and polyacrylamide are the most commonly used substrates [16–18]. Recently, we found that ECM stiffness activated the mechanosensitive ion channel Piezo1 and resulted in senescence and apoptosis of NP cells [19]. Piezo1 mediates mechanical responses in various cell types, such as osteoblasts, vascular endothelial cells, and epithelial cells [20–22]. However, the downstream mechanism of Piezo1 activation by matrix stiffness require further investigation.

Mitochondria play a key role in various cellular functions, including energy metabolism, calcium homeostasis, and reactive oxygen species (ROS) production [23]. Mitochondria are in a dynamic balance by the coordination of fission and fusion, known as mitochondrial dynamics [24]. Mitochondrial dynamics are regulated by a family of GTPases, in which dynamin-related protein 1 (Drp1) plays an essential role in mitochondrial fission [25]. Drp1 activity is regulated by several post-translational modifications, especially phosphorylation at S616 and S637 [26]. Furthermore, the mitochondrial dynamics were reported to be regulated by mechanical factors. For example, cyclic mechanical strain increased mitochondrial fission and ROS production in endothelial cells in a cytoskeleton-dependent manner [27]. Another study demonstrated that cyclic stretch led to mitochondrial ROS-induced lung injury during mechanical ventilation [28]. Recently, a new study revealed that matrix stiffness modulated mitochondrial shape and function in aging and cancer [29]. Our previous study found that stiff substate increased intracellular ROS levels, and we next want to investigate whether mitochondrial fission was involved in it.

In this study, we first found that stiff substrate increased mitochondrial fission in NP cells. Then we investigated the underlying mechanism of mitochondrial fission caused by substrate stiffness. Further studies revealed that stiff substrate increased the phosphorylation of Drp1 at S616 by activating the ERK1/2 pathway, which promoted mitochondrial fission and apoptosis in NP cells. Furthermore, Piezo1 activation was involved in the regulation of the post-translational modifications of Drp1 and mitochondrial fission caused by matrix stiffness.

## Materials and methods

### NP tissues collection

Human NP tissues were collected from patients who underwent spine surgery for idiopathic scoliosis or degenerative disc diseases. The grade of IDD was evaluated according to the Pfirrmann magnetic resonance imaging-grading system [30]. Pfirrmann grade I or II was classified as control group (NC), while Pfirrmann grade III or IV was classified as degenerative group (IDD). A total of 12 patients’ NP tissue samples were collected (3/grade), 7 males and 5 females aged 13–54 years. We defined 3 tissues as Grade I because of idiopathic scoliosis, while the other 9 tissues were defined as Grade II-IV because the patients had degenerative disc diseases in different segments. All experimental protocols were approved by the Ethics Committee of Tongji Medical College, Huazhong University of Science and Technology (no. S214). Written informed consent was obtained from all patients.

### NP cells isolation and culture

NP cells were isolated in accordance with a previous study [31]. Briefly, NP tissues were cut into pieces and enzymatically digested in 0.2% type II collagenase for 3 h. After washing and centrifugation, the isolated cells were cultured in Dulbecco’s Modified Eagle Medium/Nutrient Mixture F-12 (Gibco) containing 15% fetal bovine serum (Gibco). NP cells from the second passage were used for the subsequent experiments. Previous studies have shown that the elastic modulus of healthy NP tissues is 0.3–5 kPa, that of mildly degraded NP tissues is 10–17 kPa, and that of severely degraded NP tissues is 20–25 kPa [19, 32, 33]. To build the gradient stiffness model, NP cells were cultured for 48 h in 1 kPa (soft), 12 kPa (moderate) and 25 kPa (stiff) polystyrene plates (Matrigen, Brea, CA). The Matrigen plates are commercially available produces with variant stiffness and biocompatibility, which enables researchers to study matrix stiffness on cell behaviors [34, 35].

### Immunohistochemistry

NP specimens were fixed with formaldehyde, embedded in paraffin, and sliced into 4 μm sections. Then, the sections were incubated with antibodies against: Drp1 (Abcam, ab184247, 1:500), phospho-Drp1(Ser616) (CST, 3455, 1:300), phosphor-Drp1(Ser637) (CST, 4867, 1:300). Staining was performed using the Dako REAL EnVision Detection System, Peroxidase/DAB + , Rabbit/Mouse (Dako Cytomation), in accordance with the manufacturer's instructions. The sections were viewed under a microscope (Olympus).

### Western blot analysis

Cells were lysed in RIPA lysis buffer (Beyotime, China). The isolated proteins were separated by sodium dodecyl sulfate polyacrylamide gel electrophoresis and transferred onto a PVDF membrane (Millipore, USA). The membranes were blocked with 5% milk for 1 h. Primary antibodies against the following proteins were used: Drp1 (Abcam, ab184247, 1:1000), phospho-Drp1(Ser616) (CST, 3455, 1:1000), phosphor-Drp1(Ser637) (CST, 4867, 1:1000), CDK1 (Proteintech, 19532–1-AP, 1:2000), Cyclin B1 (Proteintech, 28603–1-AP, 1:1000), p-ERK1/2 (Proteintech, 28,733–1-AP, 1:1000), ERK1/2 (Proteintech, 11,257–1-AP, 1:2000), p-CAMKII (CST, 12,716, 1:1000), CAMKII (CST, 3357, 1:2000), Piezo1 (Abcam, ab128245, 1:1000), cleaved caspase-3 (CST, 9664, 1:1000), Bcl-2 (Proteintech, 12,789–1-AP, 1:2000), Bax (Proteintech, 50,599–2-Ig, 1:2000), GAPDH (Proteintech, 12,789–1-AP, 1:5000), COX IV (Proteintech, 11,242–1-AP, 1:5000). GADPH and COX IV were used for normalization.

### Immunofluorescence analysis

NP cells were fixed with 4% paraformaldehyde and permeabilized with 0.2% Triton X-100 for 30 min. The cells were washed twice in phosphate-buffered saline, blocked with 2% goat serum for 1 h, and then incubated with primary antibody against Drp1 (Abcam, ab184247, 1:100). Mitochondria were stained with 50 mM MitoTracker (Beyotime). Nuclei were stained for 5 min with DAPI (Beyotime). Immunofluorescence images were captured using a fluorescence microscope (Olympus, BX53, United States). For colocalization analysis of Drp1 and mitochondria, the Pearson coefficient was calculated using Image J (NIH) software and the Coloc 2 plugin. Quantifications of colocalization was performed in 20 cells of each group. In addition, the mitochondrial lengths were measured using available macro from NIH ImageJ software according to a previous study [36].

### RNA interfering

Small interfering RNA (siRNA) targeting Piezo1 and scrambled siRNA (si-scram) were synthesized by RiboBio (Guangzhou, China). The sequences of si-Piezo1 targeted for 5′-GGGACTGCCTCATTCTGTA-3′. NP cells were transfected with Lipofectamine 2000 (Invitrogen) according to the manufacturer’s protocol.

### Flow cytometry

Mitochondrial membrane potential, intracellular Ca^2+^ levels, and total ROS levels in human NP cells from each group were assessed using the JC-1 assay kit (Beyotime), Fluo-4 AM (MCE), and DCFH-DA (Beyotime), respectively. The experiments were performed according to the manufacturer’s protocols. For detecting intracellular Ca^2+^, Fluo-4 AM was added to the pretreated cells, then the preparation was incubated at 37 °C for 30 min. Next, cells were washed thrice with PBS before being trypsinized and resuspended in fresh PBS. For detecting total ROS levels, cells were incubated with 10 μm DCFH-DA in culture media for 30 min. Then, cells were washed with PBS, trypsinized, resuspended in fresh PBS. After labelling, the samples were examined using a flow cytometer (BD FACSCalibur; BD Biosciences, San Jose, USA).

### TUNEL staining

TUNEL staining was used to assess cell apoptosis. Cells were fixed in 4% paraformaldehyde for 30 min and treated with 0.5% TritonX-100 for 10 min. After being washed twice, the cells were incubated with the TUNEL staining kit (Beyotime, China) in accordance with the manufacturer’s protocol. Images were captured by a fluorescence microscope (Olympus, BX53, United States).

### Statistical analysis

Data are presented as the means ± SD of at least three independent experiments. Statistical analyses were performed using GraphPad Prism 8 software (La Jolla, CA, USA). Student’s t test was used to evaluate differences between two groups and one-way ANOVA was used to determine differences between multiple groups [37–39]. Statistical significance was set at P < 0.05 while P > 0.05 was considered nonsignificant (#). *P < 0.05, **P < 0.01, ***P < 0.001.

## Results

### The phosphorylation of Drp1 at S616 increased in degenerative NP tissues and NP cells cultured on stiff substrate

**Fig. 1:**
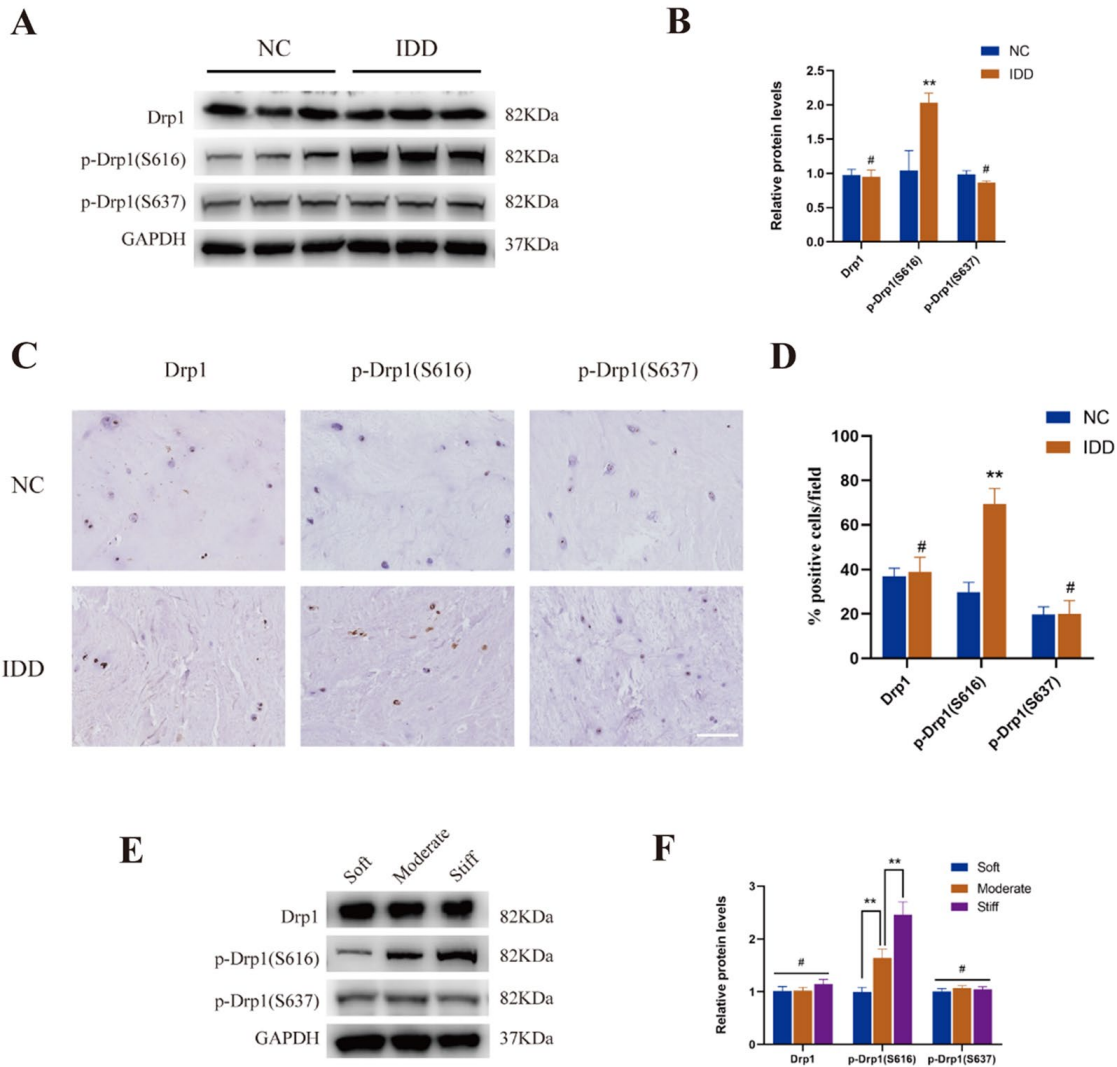
The expression of Drp1 in NP tissues and NP cells cultured on different substrate stiffness. **A, B** Protein levels and quantifications analysis of total Drp1, p-Drp1(S616) and p-Drp1(S637) in control group (NC) and degenerative group (IDD) tissues. Data were presented as the mean ± SD, n = 3. #Not significant; *P < 0.05, **P < 0.01 (Student’s t tests). **C, D** Immunochemistry staining and quantifications analysis of Drp1, p-Drp1(S616) and p-Drp1(S637) in NC and IDD tissues. Scale bar: 50 μm. Drp1 (Abcam, ab184247, 1:500), phospho-Drp1(Ser616) (CST, 3455, 1:300), phosphor-Drp1(Ser637) (CST, 4867, 1:300). Data were presented as the mean ± SD, n = 3. ^#^Not significant; *P < 0.05, **P < 0.01 (Student’s t tests). **E, F** Protein levels and quantifications analysis of total Drp1, p-Drp1(S616) and p-Drp1(S637) in NP cells cultured on different substrate stiffness. Data were presented as the mean ± SD, n = 3. ^#^Not significant; *P < 0.05, **P < 0.01, ***P < 0.001 (one-way ANOVA)

To observe the roles of Drp1 in IDD, we measured the expression of total Drp1, p-Drp1(S616) and p-Drp1(S637) in NP tissues of control group (NC) and degenerative group (IDD). As shown in Fig. 1A, B, the level of Drp1 phosphorylated at Ser616 significantly increased in degenerative NP tissues, while the total Drp1 protein and phosphorylation of Drp1 at Ser637 showed no significant change. Furthermore, immunohistochemistry also showed an increase expression of Drp1 phosphorylated at Ser616 in the degenerative NP tissues, while no obvious change was detected in the level of total Drp1 and Drp1 phosphorylated at Ser637 (Fig. 1C, D). We then investigated whether the expression of Drp1 were regulated by matrix stiffness. According to the stiffness of NP tissues at different degrees of degeneration, NP cells were cultured on polystyrene plates with different stiffness ranging from 1 to 25 kPa. We found that the effect of matrix stiffness on Drp1 expression was similar to that found in degenerative NP tissues. As shown in Fig. 1E, F, the expression of Drp1 phosphorylated at Ser616 was increased when NP cells were cultured on stiff substrate, while the total Drp1 protein levels and Drp1 phosphorylated at Ser637 had no significant changes. Together, these findings indicated that the phosphorylation of Drp1 at S616 increased in degenerative NP tissues and NP cells cultured on stiff substrate.

### Stiff ECM increased the phosphorylation of Drp1 at S616 by activating the ERK1/2 pathway

**Fig. 2:**
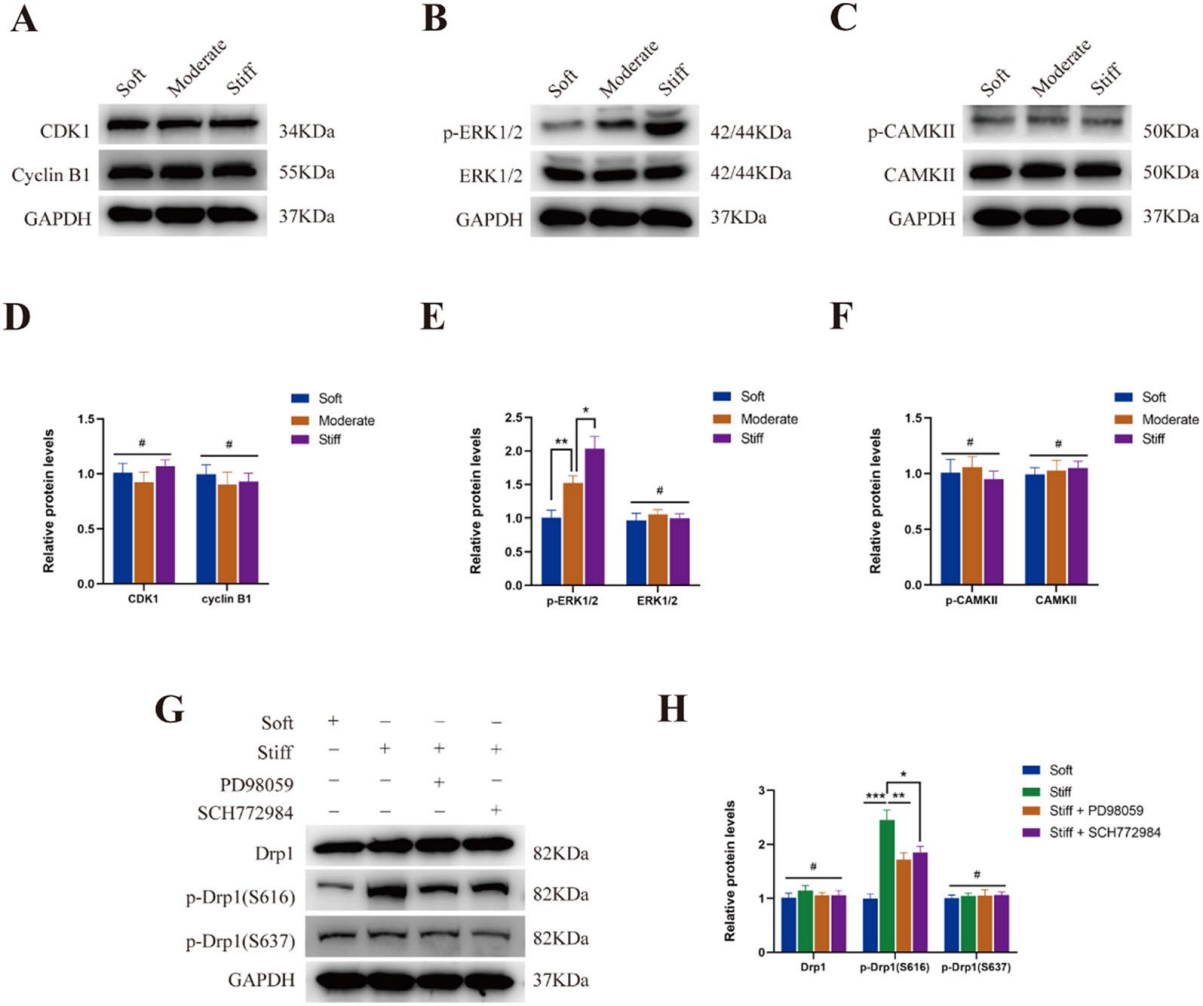
Stiff ECM increased the phosphorylation of Drp1 at S616 by activating the ERK1/2 pathway. **A**–**D** Protein levels and quantifications analysis of CDK1 and Cyclin B1 in NP cells cultured on different substrate stiffness. Data were presented as the mean ± SD, n = 3. ^#^Not significant; *P < 0.05 (one-way ANOVA). **B**–**E** Protein levels and quantifications analysis of p-ERK1/2 and ERK1/2 in NP cells cultured on different substrate stiffness. Data were presented as the mean ± SD, n = 3. #Not significant; *P < 0.05, **P < 0.01, ***P < 0.001 (Student’s t tests for two groups and one-way ANOVA for multiple groups). **C**–**F** Protein levels and quantifications analysis of p-CAMKII and CAMKII in NP cells cultured on different substrate stiffness. Data were presented as the mean ± SD, n = 3. #Not significant; *P < 0.05 (one-way ANOVA). **G**, **H** Protein levels and quantifications analysis of total Drp1, p-Drp1(S616) and p-Drp1(S637) in different groups. Data were presented as the mean ± SD, n = 3. #Not significant; *P < 0.05, **P < 0.01, ***P < 0.001 (Student’s t tests for two groups and one-way ANOVA for multiple groups)

According to previous studies, phosphorylation of Ser616 in the variable domain has generally been shown to result in activation of Drp1, and it is targeted by a variety of upstream kinases, including cyclin-dependent kinase 1/cyclin B1 (CDK1/Cyclin B1), extracellular signal-regulated kinase 1/2 (ERK1/2), and calmodulin-dependent protein kinase II (CaMKII) signaling pathway [40–42]. To further investigate the effect of matrix stiffness on Drp1 expression, the protein expression levels of CDK1/Cyclin B1, p-ERK1/2 / ERK1/2, and p-CaMKII/ CaMKII were examined in NP cells cultured on different substrates. As shown in Fig. 2A–F, the expression of p-ERK1/2 was increased when NP cells were cultured on stiff substrate, while the expression of CDK1/Cyclin B1 and p-CaMKII/ CaMKII had no significant changes. To further confirm the role of ERK1/2 in the regulation of Drp1 phosphorylation, the small molecule inhibitors PD98059 (10 μM, MCE) and SCH772984 (10 μM, MCE) were used to suppress ERK1/2 pathway. As shown in Fig. 2G–H, the treatment of PD98059 and SCH772984 can reverse the increase of Drp1 phosphorylation at Ser616 caused by substrate stiffness in NP cells. These results indicated stiff ECM increased the phosphorylation of Drp1 at S616 by activating the ERK1/2 pathway in NP cells.

### Stiff ECM increased mitochondrial fission by enhancing the recruitment of Drp1 to mitochondria

**Fig. 3:**
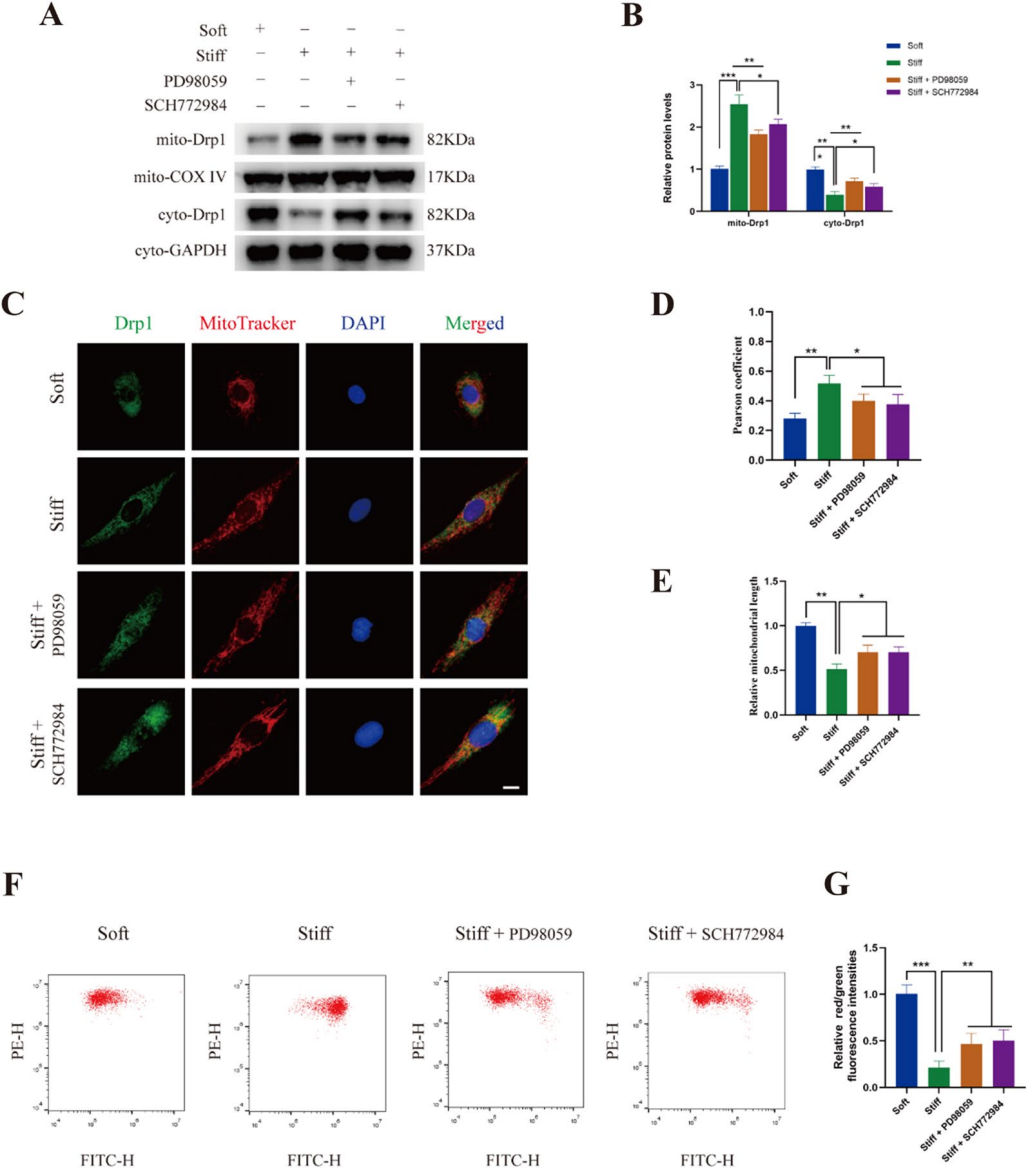
Stiff ECM increased mitochondrial fission by enhancing the recruitment of Drp1 to mitochondria. **A**, **B** The protein levels of mitochondrial Drp1 and cytoplasmic Drp1 in NP cells were measured by western blot. Data were presented as the mean ± SD, n = 3. #Not significant; *P < 0.05, **P < 0.01, ***P < 0.001 (Student’s t tests). **C, D** The colocalization of Drp1 (Green) and Mitotracker (Red) was examined by immunofluorescence in NP cells of Soft group, Stiff group, and Stiff substrate with PD98059 or SCH772984 treatment group. Quantifications of colocalization was performed in 20 cells of each group. Scale bar: 10 μm. Data were presented as the mean ± SD. #Not significant; *P < 0.05, **P < 0.01, ***P < 0.001 (Student’s t tests). **E** Quantifications of mitochondrial length in Soft group, Stiff group, and Stiff substrate with PD98059 or SCH772984 treatment group (20 cells/group). Data were presented as the mean ± SD. #Not significant; *P < 0.05, **P < 0.01, ***P < 0.001 (Student’s t tests). **F**, **G** Mitochondrial membrane potential was detected by JC-1 staining and measured by flow cytometry. Data were presented as the mean ± SD, n = 6. #Not significant; *P < 0.05, **P < 0.01, ***P < 0.001 (Student’s t tests)

As post-translational modifications of Drp1 have a significant effect on mitochondrial dynamics, based on the findings above, we hypothesized that stiff substrate increased mitochondrial fission. Migration of Drp1 from the cytoplasm to mitochondria is a prerequisite for mitochondrial fission. As shown in Fig. 3A, B, stiff ECM resulted in an increase in the mitochondrial Drp1 level, accompanied by decreased cytosolic levels. Immunofluorescence staining showed a stronger colocalization of Drp1 with mitochondria in the stiff substrate group, confirming the translocation of Drp1 to the mitochondria (Fig. 3C, D). A shortened mitochondrial length was found in NP cells cultured on the stiff substrate (Fig. 3E). We found that PD98059 and SCH772984 partly reversed the recruitment of Drp1 to the mitochondria induced by matrix stiffness. Furthermore, flow cytometry revealed that stiff substrate significantly increased the mitochondrial membrane potential, which can be abolished by small molecule inhibitors of ERK1/2 (Fig. 3F, G). Collectively, these results suggested that stiff substrate enhanced mitochondrial fission by promoting the recruitment of Drp1 to the mitochondria.

### Piezo1 activity mediated the post-translational modifications of Drp1 caused by matrix stiffness

**Fig. 4:**
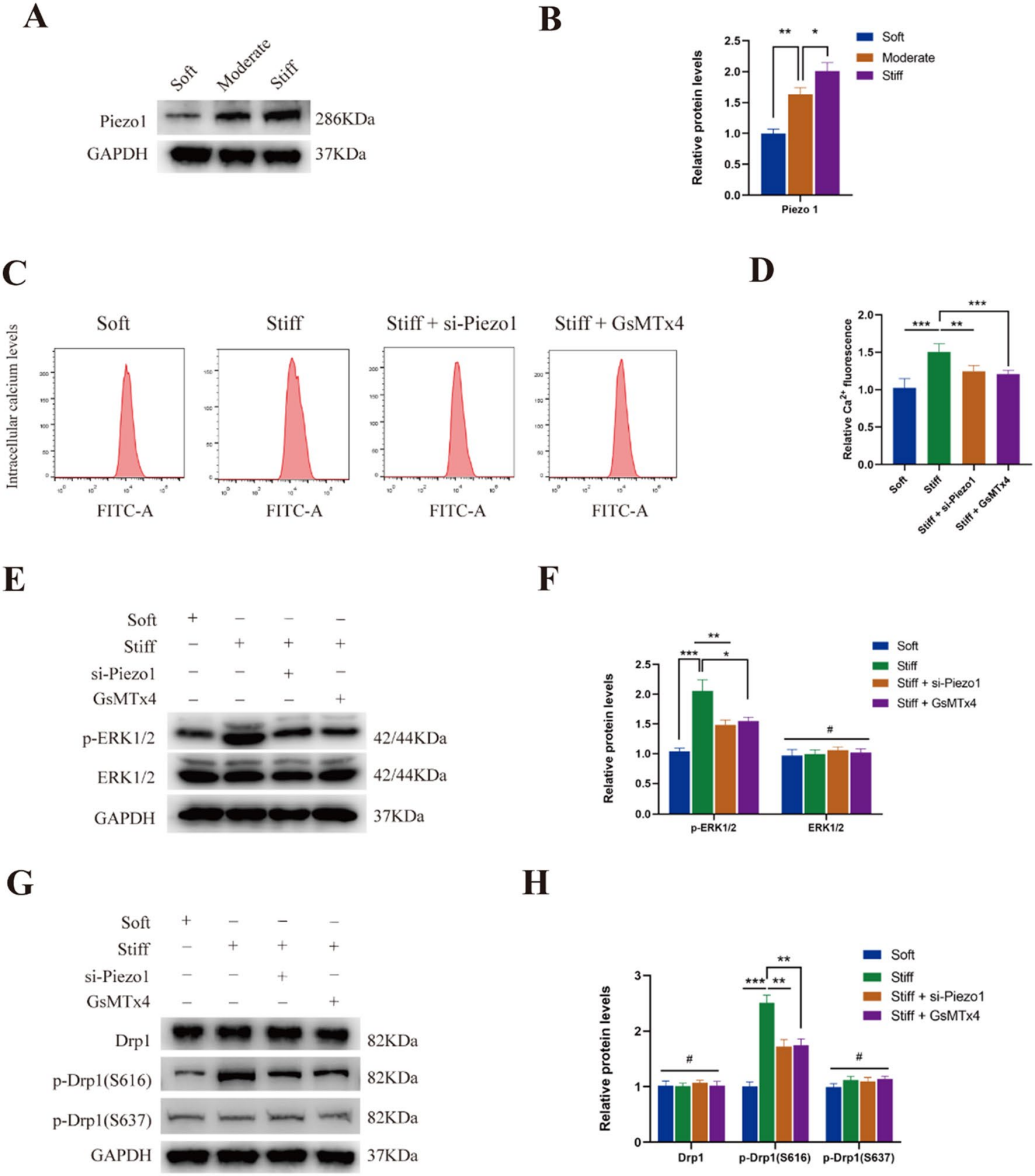
Piezo1 activity mediated the post-translational modifications of Drp1 caused by matrix stiffness. **A, B** The protein level of Piezo1was measured by western blotting in NP cells cultured on different substrate stiffness. Data were presented as the mean ± SD, n = 3. #Not significant; *P < 0.05, **P < 0.01, ***P < 0.001 (Student’s t tests). **C, D** Intracellular Ca^2+^ levels measured by flow cytometry in Soft group, Stiff group, and Stiff substrate with si-Piezo1 or GsMTx4 treatment group. Data were presented as the mean ± SD, n = 6. #Not significant; *P < 0.05, **P < 0.01, ***P < 0.001 (Student’s t tests). **E, F** Protein levels and quantifications analysis of p-ERK1/2 and ERK1/2 in Soft group, Stiff group, and Stiff substrate with si-Piezo1 or GsMTx4 treatment group. Data were presented as the mean ± SD, n = 3. #Not significant; *P < 0.05, **P < 0.01, ***P < 0.001 (Student’s t tests for two groups and one-way ANOVA for multiple groups). **G**, **H** Protein levels and quantifications analysis of total Drp1, p-Drp1(S616) and p-Drp1(S637) in Soft group, Stiff group, and Stiff substrate with si-Piezo1 or GsMTx4 treatment group. Data were presented as the mean ± SD, n = 3. #Not significant; *P < 0.05, **P < 0.01, ***P < 0.001 (Student’s t tests for two groups and one-way ANOVA for multiple groups)

Piezo1 mediates mechanical responses in various cell types, such as osteoblasts, vascular endothelial cells, and epithelial cells. The mechanosensitive Piezo1 is a Ca^2+^ channel that responds to different mechanical stimulations. Therefore, we examined the influence of substrate stiffness on Piezo1 expression in NP cells. The results revealed that stiff substrate increased the expression of Piezo1 (Fig. 4A, B). As shown in Fig. 4C, D, stiff substrate triggered an increase in intracellular Ca^2+^ levels, which could be suppressed by Piezo1 silencing or GsMTx4 (an inhibitor of Piezo1, 5 μM, MCE). Next, we investigated whether the activation of ERK1/2 pathway and phosphorylation of Drp1 caused by matrix stiffness were mediated by Piezo1 activity. As shown in Fig. 4E, F, Piezo1 silencing and GsMTx4 suppressed the increase of p-ERK1/2 caused by matrix stiffness in NP cells. According to previous studies, the actomyosin cytoskeleton and RhoA/ROCK signalling pathway play a leading role in the response to mechanical stimuli [43–46]. To further clarify the signaling cascade leading to ERK1/2 activation, NP cells were exposed to Blebbistatin (an inhibitor of myosin II, 10 μM, MCE), and Y-27632 (an inhibitor of ROCK, 10 μM, MCE). As shown in Additional file 1: Fig. S1, phosphorylation of ERK1/2 was not affected by Blebbistatin or Y-27632, which suggested that myosin II and RhoA/ROCK signalling pathway were not involved in stiff substrate-induced ERK1/2 activation. Moreover, the increase in Drp1 phosphorylated at Ser616 was abolished by Piezo1 inhibition, while the total Drp1 protein levels and phosphorylation at Ser637 were not affected by Piezo1 activity (Fig. 4G, H). These results indicated that the activation of ERK1/2 pathway and phosphorylation of Drp1 caused by matrix stiffness were mediated by Piezo1 activity.

### Piezo1 inhibition attenuated matrix stiffness-induced mitochondrial fission

**Fig. 5:**
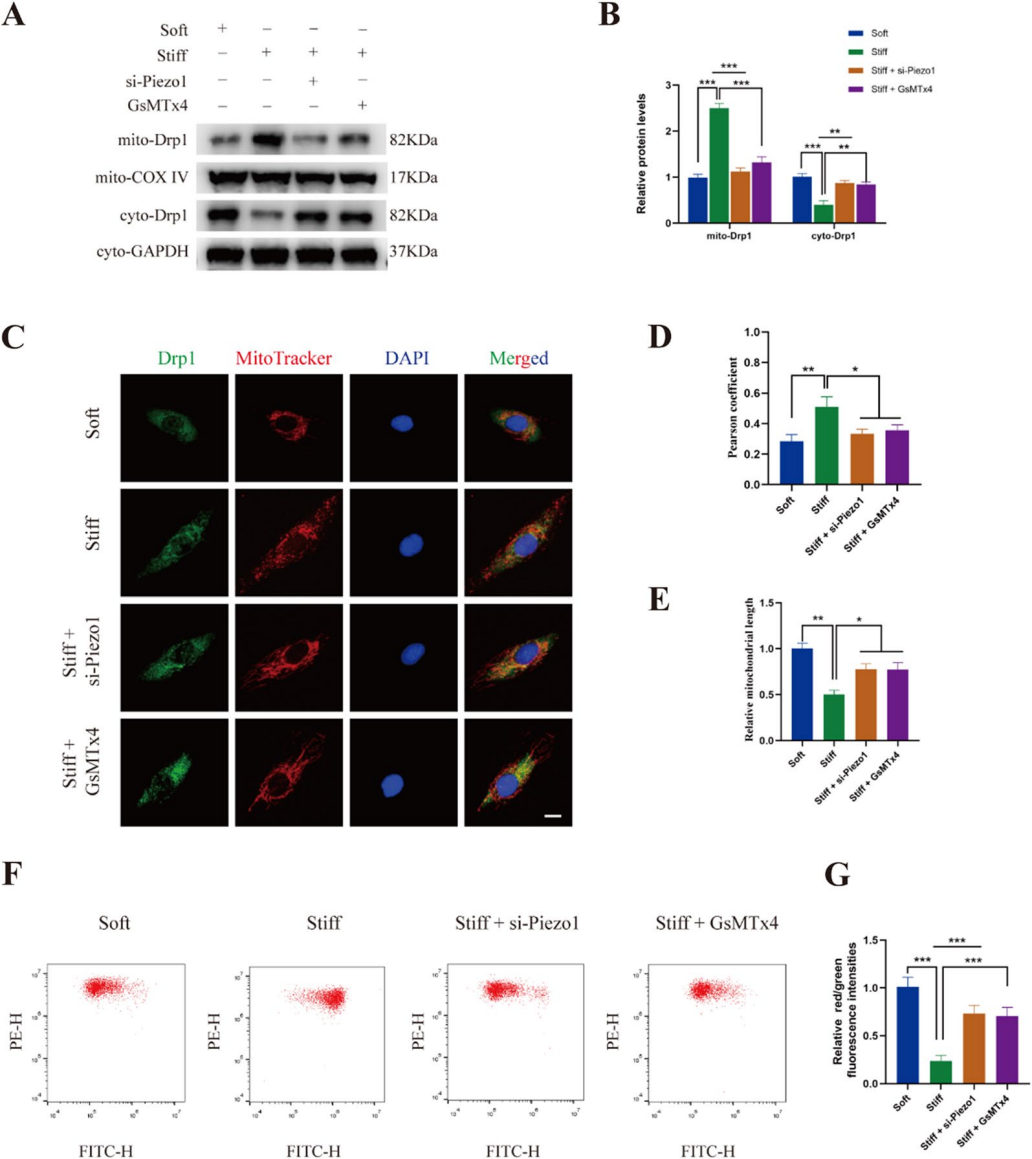
Piezo1 inhibition attenuated matrix stiffness-induced mitochondrial fission. **A, B** The protein levels of mitochondrial Drp1 and cytoplasmic Drp1 in different group were measured by western blot. Data were presented as the mean ± SD, n = 3. #Not significant; *P < 0.05, **P < 0.01, ***P < 0.001 (Student’s t tests). **C, D** The colocalization of Drp1 (Green) and Mitotracker (Red) was examined by immunofluorescence in different group. Quantifications of colocalization was performed in 20 cells of each group. Scale bar: 10 μm. Data were presented as the mean ± SD. #Not significant; *P < 0.05, **P < 0.01, ***P < 0.001 (Student’s t tests). **E** Quantifications of mitochondrial length in different group (20 cells/group). Data were presented as the mean ± SD. #Not significant; *P < 0.05, **P < 0.01, ***P < 0.001 (Student’s t tests). **F**, **G** Mitochondrial membrane potential was detected by JC-1 staining and measured by flow cytometry in different group. Data were presented as the mean ± SD, n = 6. #Not significant; *P < 0.05, **P < 0.01, ***P < 0.001 (Student’s t tests)

We next investigated whether Piezo1 activity regulated Drp1 localization and mitochondrial fission. As shown in Fig. 5A, B, Piezo1 inhibition by knock down or GsMTx4 both reversed the recruitment of Drp1 to mitochondria induced by stiff substrate. Immunofluorescence staining confirmed the suppression of Drp1 translocation to the mitochondria by Piezo1 inhibition (Fig. 5C, D). Piezo1 inhibition also reversed the mitochondrial fission caused by substrate stiffness (Fig. 5E). Furthermore, flow cytometry demonstrated that the increase of the mitochondrial membrane potential caused by stiff substrate was abolished by Piezo1 silencing or GsMTx4 (Fig. 5F, G). These results revealed that matrix stiffness-induced mitochondrial fission can be suppressed by blocking Piezo1 activity.

### Piezo1-mediated mitochondrial fission was involved in matrix stiffness-induced apoptosis of NP cells

**Fig. 6:**
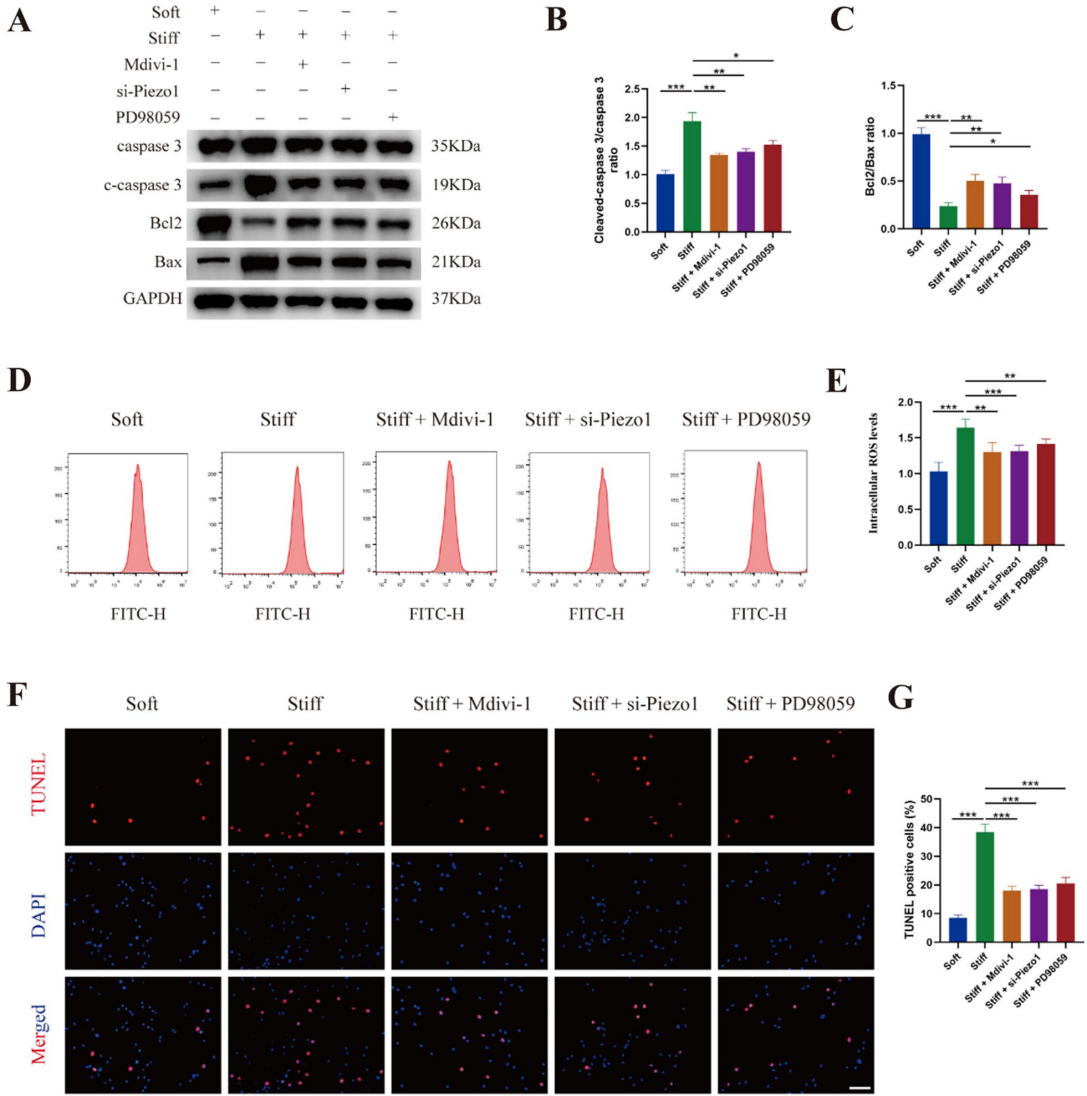
Piezo1-mediated mitochondrial fission was involved in matrix stiffness-induced apoptosis of NP cells. **A**–**C** The protein levels and quantifications analysis of caspase, c-caspase, Bcl-2, and Bax in different group were measured by western blot. Data were presented as the mean ± SD, n = 3. ^#^Not significant; *P < 0.05, **P < 0.01, ***P < 0.001 (Student’s t tests). **D**, **E** The ROS levels were measured using DCFH-DA and analyzed by a flow cytometer. Data were presented as the mean ± SD, n = 6. ^#^Not significant; *P < 0.05, **P < 0.01, ***P < 0.001 (Student’s t tests). **F**, **G** TUNEL analysis and corresponding quantification of cell apoptosis in different group. Scale bar: 60 μm. Data were presented as the mean ± SD, n = 3. ^#^Not significant; *P < 0.05, **P < 0.01, ***P < 0.001 (Student’s t tests)

Our previous study found that stiff substrate increased reactive oxygen species (ROS) level and apoptosis in NP cells, we next investigated whether mitochondrial fission was involved. As shown in Fig. 6A–C, the treatment of Mdivi-1 (a blocker of mitochondrial fission, 20 μM, MCE), Piezo1 silencing and PD98059 significantly reversed the increase in cleaved-caspase 3 and the decrease in Bcl-2/Bax. The increased ROS levels caused by stiff substrate were also inhibited by Mdivi-1, Piezo1 silencing and PD98059 in NP cells (Fig. 6D, E). Furthermore, TUNEL staining revealed that Mdivi-1, Piezo1 silencing and PD98059 were efficient to attenuate stiff substrate-induced apoptosis in NP cells (Fig. 6F, G). Overall, these results suggested that Piezo1-mediated mitochondrial fission was involved in matrix stiffness-induced apoptosis in NP cells.

## Discussion

**Fig. 7:**
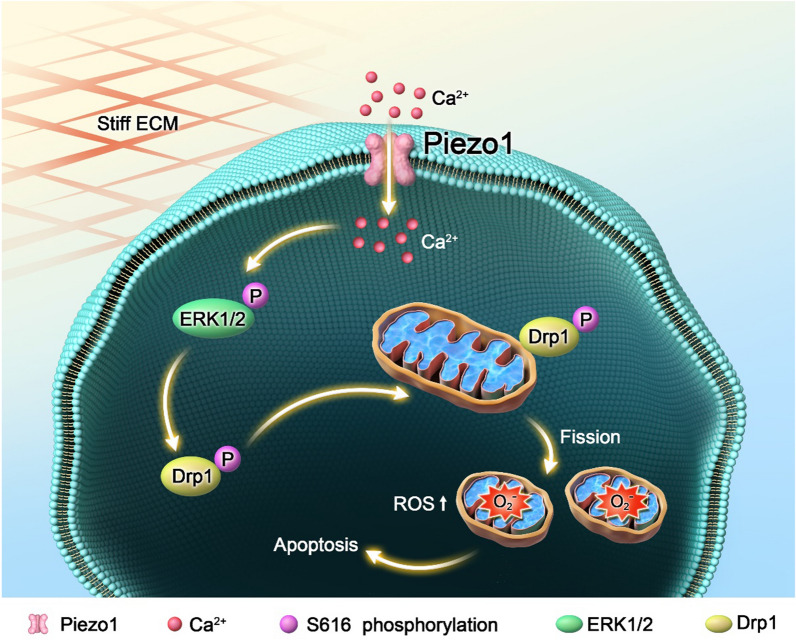
Schematic graph of matrix stiffness induced mitochondrial fission through Piezo1 in human intervertebral disc degeneration. Stiff ECM caused Piezo1 activation and Ca^2+^ influx, result in ERK1/2 activation and phosphorylation of Drp1 at S616, and finally lead to mitochondrial fission and apoptosis in NP cells

Recently, the mechanisms of mechanical stimulation in the development of different diseases have attracted significant attention. Various studies on different cell types have shown that mechanical cues in the microenvironment can significantly regulate cell proliferation, migration, and phenotypic changes [32, 47, 48]. Previous studies on NP cells showed that soft substrate maintained a juvenile-like state of NP cells, while stiff substrate induced a degenerative-like phenotype [32, 49]. Recently, we found that ECM stiffness activated the mechanosensitive ion channel Piezo1 and resulted in senescence and apoptosis of NP cells [19]. The major finding of this study was that the apoptosis of NP cells on stiff substrate was induced by Piezo1-mediated phosphorylation of Drp1 and mitochondrial fission (Fig. 7). This study revealed a new mechanism of mechanotransduction in NP cells, contributing to our understanding of the molecular mechanisms underlying IDD and the potential role of mechanosensitive ion channels in NP cell responses to mechanical cues, providing novel insights into the development of therapies for treating IDD.

Extracellular matrix stiffness is emerging as a crucial mechanical cue that drives the progression of various diseases, such as cancer, fibrosis, and inflammation [50]. Cells respond to ECM stiffness by activating multiple downstream signaling pathways. For example, stiff substrate can promote focal adhesion kinase activation and actomyosin cytoskeletal remodeling [51, 52], or it can trigger the translation of multiple transcription factors to enter the nucleus, such as myocardin-related transcription factor–A (MRTF-A) and yes-associated protein/PDZ-binding motif (YAP/TAZ) [32, 53]. Furthermore, the mechanosensitive ion channel Piezo1 plays an important role in the response to mechanical stimulations [20–22]. Piezo1 mediates a variety of biological processes, such as bone development, blood pressure regulation, and immune response [20, 54, 55]. In NP cells, Piezo1 was reported to be activated by mechanical stretch stimulation and induced the activation of the NLRP3 inflammasome [56]. Our previous study found that stiff substate induced the activation of the Piezo1 channel and increased Ca^2+^ influx. In this study, our further investigations suggested that activated Piezo1 increased phosphorylation of Drp1 at S616, and thus promoted mitochondrial fission and apoptosis in NP cells. In addition, inhibition of mitochondrial division by Mdivi-1 or Piezo1 knockdown can effectively alleviate stiffness-induced apoptosis in NP cells.

Balanced mitochondrial dynamics are essential for maintaining normal cellular function. Under normal physiological conditions, mitochondria provide energy for cells through the dynamic conversion of fusion and fission [57]. However, excessive mitochondrial fission leads to increased ROS production and inflammatory responses, ultimately leading to cell death [58]. Previous studies have mainly focused on the effects of various biochemical stimuli on mitochondrial dynamics [59–61]. Recently, several studies reported that mitochondria respond not only to biochemical stimuli, but also to mechanical cues. For example, mechanical stimulus can be sensed by the mitochondrial fission factor (MFF), causing Drp1 recruitment and mitochondrial fission [62]. Another investigation revealed that actin filaments can directly bind Drp1 and increase GTPase activity, ultimately increasing mitochondrial fission [63]. In NP cells, compression stimulation was reported to enhance mitochondrial fission by decreasing the expression of the mitochondrial fusion proteins and increasing the expression of Drp1 [64]. In this study, we first found that stiff substrate increased mitochondrial fission by triggering the translocation of Drp1 to the mitochondria in NP cells. Stiff ECM triggered the activation of ERK1/2 pathway, and increased the phosphorylation of Drp1. We further found that Piezo1 knockdown suppressed the ERK1/2 pathway and Drp1 phosphorylation, and reversed the recruitment of Drp1 to mitochondria. These results revealed that Piezo1 activity mediated the post-translational modifications of Drp1 and mitochondrial fission caused by matrix stiffness.

Piezo1 activation causes Ca^2+^ influx, which has extensive effects on downstream signaling pathways [65]. Increased intracellular Ca^2+^ can phosphorylate or dephosphorylate the target protein. Wu et al. demonstrated that increased Ca^2+^ influx triggered the ERK1/2-mediated phosphorylation at S616 or calmodulin-binding catalytic subunit A-mediated dephosphorylation at S637 in diabetic cardiomyopathy [40]. It has been reported that activated Piezo1 phosphorylated the target protein by increasing the expression of calcium/calmodulin-dependent protein kinase II [66, 67]. Piezo1-mediated mechanotransduction was also reported to activate calcineurin signaling and dephosphorylated the downstream proteins [68]. In this study, we found that Piezo1 activation-mediated Ca^2+^ influx was involved in the regulation of Drp1 phosphorylation in NP cells caused by matrix stiffness. Intracellular Ca^2+^ overload was reported to induce Drp1 phosphorylation by several ways, such as CDK1/Cyclin B1, ERK1/2, and CaMKII signaling pathway. Our results demonstrated that stiff substrate increased the phosphorylation of Drp1 at S616 by activating the ERK1/2 pathway in NP cells, while CDK1/Cyclin B1 and CaMKII signaling pathway had no significant change. This difference may be due to different stimulation and cell types and need to be further investigated.

The present study has some limitations. First, the NP cells were cultured on the surface of substrates rather than 3D culture, which cannot fully represent in vivo conditions. Recently, researchers tend to culture cells in 3D environments to simulated internal environment. Some studies showed that 2D and 3D culture had similar effects, while others revealed that 3D cultures had different effects on cell behavior. Therefore, the findings of this study in 2D culture need to be further verified in 3D culture. Second, the post-translational modification of Drp1 included several types, and we only studied Drp1 phosphorylation in this study. For example, Drp1 can be phosphorylated at N-terminal GTPase domain sites Ser40/44 or SUMO-ylation at Lys532, 535, 558 and 568, which may play a key role in IDD. In addition, the NP tissues of the control group were obtained from the patients with idiopathic scoliosis, which may not fully represent the normal NP tissues. Therefore, further investigations are needed to confirm the results of this study.

## Conclusion

In summary, our study revealed that stiff substrate increased the phosphorylation of Drp1 at S616 by activating ERK1/2 pathway, which promoted mitochondrial fission and apoptosis in NP cells. Furthermore, Piezo1 activation was involved in the regulation of the post-translational modifications of Drp1 and mitochondrial fission caused by matrix stiffness. Furthermore, further investigations using 3D culture models to better mimic the in vivo microenvironment and exploring the relevance of other post-translational modifications of Drp1 are needed in the future. The identification of Piezo1 as a key player in regulating the post-translational modifications of Drp1 and mitochondrial fission in response to matrix stiffness represents a significant contribution to the field of mechanobiology and can be used for biomedical applications. The novel insights into the interplay between mechanical cues, intracellular signaling pathways, and cellular responses open new grounds for understanding the pathogenesis of IDD and may have broader implications in studying other diseases influenced by substrate stiffness.

### Supplementary Information

**Additional file 1: Figure S1.** Protein levels and quantifications analysis of p-ERK1/2 and ERK1/2 in different groups. Data were presented as the mean ± SD, n = 3. #Not significant; *P < 0.05, **P < 0.01, ***P < 0.001 (Student’s t tests for two groups and one-way ANOVA for multiple groups).

## Data Availability

All data generated or analyzed during this study are included in this article. Further enquiries can be directed to the corresponding author.

## Abbreviations

Drp1: Dynamin-related protein 1
ECM: Extracellular matrix
ERK1/2: Extracellular signal-regulated kinase 1/2
IDD: Intervertebral disc degeneration
LBP: Low back pain
NP: Nucleus pulposus
ROS: Reactive oxygen species
