# Supplementary material for: Matrix stiffness induces Drp1-mediated mitochondrial fission through Piezo1 mechanotransduction in human intervertebral disc degeneration
Source: J Transl Med. 2023 Oct 10;21:711. doi: 10.1186/s12967-023-04590-w (PMC10563269; doi:10.1186/s12967-023-04590-w)
Supplement: Supplementary file 1 — Additional file 1: Figure S1. Protein levels and quantifications analysis of p-ERK1/2 and ERK1/2 in different groups. Data were presented as the mean ± SD, n = 3. #Not significant; *P < 0.05, **P < 0.01, ***P < 0.001 (Student’s t tests for two groups and one-way ANOVA for multiple groups). [file 12967_2023_4590_MOESM1_ESM.docx]

**Matrix stiffness induces Drp1-mediated mitochondrial fission through Piezo1 mechanotransduction in human intervertebral disc degeneration**


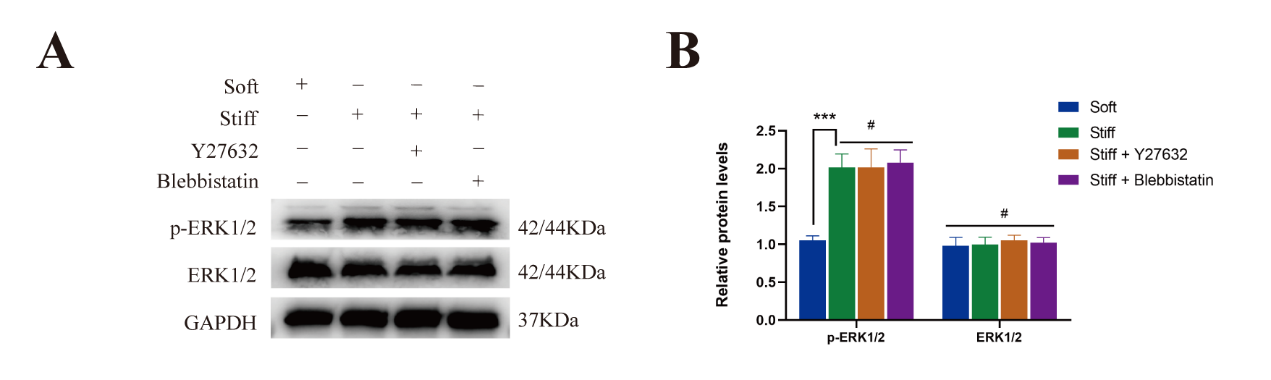


**Additional file 1: Figure S1.** Protein levels and quantifications analysis of p-ERK1/2 and ERK1/2 in different groups. Data were presented as the mean ± SD, n = 3. #Not significant; *P < 0.05, **P < 0.01, ***P < 0.001 (Student’s t tests for two groups and one-way ANOVA for multiple groups).
